# Athletes Perceived Level of Risk Associated with Botanical Food Supplement Use and Their Sources of Information

**DOI:** 10.3390/ijerph20136244

**Published:** 2023-06-28

**Authors:** Bridin McDaid, Floris C. Wardenaar, Jayne V. Woodside, Charlotte E. Neville, David Tobin, Sharon Madigan, Anne P. Nugent

**Affiliations:** 1 School of Biological Sciences, Institute for Global Food Security, Queen’s University Belfast, Belfast BT9 5DL, UK; 2 College of Health Solutions, Arizona State University, Phoenix, AZ 85004, USA; floris.wardenaar@asu.edu; 3 Centre for Public Health, Institute for Global Food Security, Queen’s University Belfast, Belfast BT9 7BL, UK; 4 Sport Ireland Institute, National Sport Campus, Abbottstown, D15 Y52H Dublin, Irelandsmadigan@instituteofsport.ie (S.M.); 5 Department of Physical Education & Sport Sciences, Faculty of Education and Health Sciences, University of Limerick, V94 T9PX Limerick, Ireland; 6 Health Research Institute, University of Limerick, V94 T9PX Limerick, Ireland; 7 Institute of Food and Health, School of Agriculture and Food Science, University College Dublin, D04 V1W8 Dublin, Ireland

**Keywords:** herbal, bioactive substances, athletes, doping, sources of information, nutrition knowledge

## Abstract

Athletes should carefully consider the use of botanical food supplements (BFSs) given the current lack of substantiation for botanical nutrition and health claims under EU and UK food laws. In addition, athletes may be at an increased risk of doping violations and other adverse outcomes potentially associated with BFS use; however, little is known about athletes’ intake, knowledge, or perceptions in relation to BFS use. An online cross-sectional survey of *n* = 217 elite and amateur athletes living on the island of Ireland was conducted using Qualtrics XM to assess intake, knowledge, attitudes, and perceptions. General food supplements (FSs) were reported by approximately 60% of the study cohort, and 16% of the supplements reported were categorized as BFS. The most frequently consumed BFSs were turmeric/curcumin (14%), Ashwagandha (10%), and Beetroot extract (8%). A higher proportion of amateur athletes would source information about BFSs from less credible sources, such as fellow athletes, or from internet sources or their coach, compared to elite athletes. Those who sourced information about botanicals from fellow athletes (*p* = 0.03) or the internet (*p* = 0.02) reported a lower perceived level of risks associated with BFS use. This study therefore suggests that amateur athletes may be more likely to source information from less credible sources compared to elite athletes who may have more access to nutrition professionals and their knowledge/advice. This may have potential adverse implications for amateur athletes, e.g., Gaelic games players, who are included within the doping testing pool but who may not have access to evidence-based nutrition advice.

## 1. Introduction

Multiple terms and definitions exist for what will be described in this article as “food supplements”. FSs, as defined under UK and EU law, are “foodstuffs the purpose of which is to supplement the normal diet and which are concentrated sources of nutrients or other substances with a nutritional or physiological effect, alone or in combination, marketed in dose form, namely forms such as capsules, pastilles, tablets, pills and other similar forms, sachets of powder, ampoules of liquids, drop dispensing bottles, and other similar forms of liquids and powders designed to be taken in measured small unit quantities” [1]. The research outlined in this article was conducted across Northern Ireland (UK) and the Republic of Ireland (EU) and thus the term “food supplement” was used as this is how they are advertised and sold to consumers in these jurisdictions. Care must be taken when athletes use FSs, especially among elite athletes who fall within the doping testing pool, i.e., those who compete in sports at the national and international level. Intake among such athletes may lead to unintentional doping through potentially being misinformed by product labels and thus consuming products with banned substances or levels of substances beyond what is set out in anti-doping regulations [2]. 

The present analysis investigated the use of both FSs and botanical food supplements (BFSs) as separate product types. Athletes have been found to consume BFSs with little further information into any specific types of botanicals consumed, as they are usually grouped together under one overarching type, such as “herbs” or “botanicals”. Some BFSs, however, are sold as testosterone boosters [3], such as Fenugreek, Ashwagandha root [4,5], and *Tribulus terrestris*, [3]. Thus, the purpose of highlighting BFSs separately was to highlight differences in the perception of risks associated with the use of BFSs between amateur athletes and elite athletes who use BFSs and those who do not. An additional reason for making this distinction is that FS use in general is well-quantified in athletic populations, with results from various studies indicating 40–100% of athletes use them [6,7], whereas botanical food supplement (BFS) intake is less well-known and, in addition, information on athletes’ knowledge, attitudes, and perceptions of BFSs is scarce. Where BFS intake has been documented among athletes, they are often grouped together as “botanicals” or “herbals”, and the specific types of BFSs used are rarely teased out and reported [8,9,10,11]. There is currently no legal, standalone definition for BFSs under food law or in nutrition practice; however, they are considered in this instance to be FSs which contain ‘botanical preparations’. Botanical preparations are outlined in EU food law by the European Commission (EC), which gives further information about the substances included in BFS products. This definition is as follows: “Preparations obtained from botanicals that may be whole or cut plant parts which have been processed in some way (i.e., pressing, squeezing, extraction, drying, fermentation etc.) and includes comminuted or powdered plants, plant parts, algae, fungi, lichen, tinctures, extracts, essential oils, expressed juices and processed exudates” [12].

BFS products are often marketed as being “natural/from natural sources”, terms which have been found to be important to consumers. A systematic review of studies which investigated food naturalness found that naturalness of food is highly important to consumers and influences their food intake significantly, but definitions for what ‘naturalness’ means differ between studies [13]. Therefore, there may be a belief among consumers of BFSs that these products are better, even though their nutrition and health claims have not been scientifically evaluated as accurate.

A European scientific consortium known as PlantLIBRA was set up to investigate population intakes of what was referred to as “plant food supplements” and developed an adapted version of the definition for FSs under EU food law to define this term. This pan-European study found that 18% of the study cohort across 6 European countries were BFS consumers and the prevalence of intake was higher among those who were women, over the age of 50 years, and who had achieved a high education level [13]. This study did not specifically investigate the athletic population for use of plant food supplements, but it indicated that older adults, who are known frequent consumers of FSs, also consumed botanicals. Thus, further investigations into the use of BFSs among known FS consumers, including athletes, are warranted, especially given the potential risks which may arise with use. Some of potential risks associated with BFS use include adverse side effects, polypharmacy-related interactions, and, for athletes, doping risks.

The risk of adverse effects such as being misled, unintentional doping, and potential adverse side effects among the athletic population should be considered by BFS users, as BFS products and their associated nutrition and health claims are not substantiated under EU/UK law, yet they are still permitted for sale on the market [12,14]. This may expose consumers to additional risks which can arise due to the lack of substantiation of such claims, e.g., being potentially misled by inaccurate on-pack labelling, adverse side effects due to interactions, and, for athletes, potential doping violations. Although these risks also exist for athletes consuming other FSs, these circumstances surrounding the lack of information on the use of BFSs by athletes and the impasse surrounding the substantiation of nutrition and health claims relating to BFSs [15] highlight a potential need to investigate the use of BFSs as well as to characterize athletes’ knowledge, attitudes, and perceptions associated with their use. In the USA, dietary supplements include botanical food supplements and are regulated by the 1994 Dietary Supplement Health and Education Act and more recently the 2011 Food Safety Modernization Act, by the Food and Drug Administration (FDA). Current regulations do not require dietary supplements, including botanical supplements, to undergo the same rigorous testing for effectiveness, interaction, or safety requirements as conventional drugs do. Approval from the FDA is not required before marketing dietary supplements in the United States.

This paper therefore aims to quantify and characterize FS and BFS intake among elite and amateur athletes living on the island of Ireland and will examine the sources of information and consultation practices employed by these athletes. This paper will uniquely investigate the differences in athletes’ perception of the risks and types of risks associated with the use of BFSs and how this may be influenced by the types of information sources being utilized. 

## 2. Materials and Methods

### 2.1. Study Design and Population

An online survey was used to collect data from elite and amateur athletes living on the island of Ireland between December 2021 and May 2022. Ethical approval for the survey was granted by Queen’s University Belfast Research and Ethics Committee for the Faculty of Medicine and Life Sciences. The survey duration was 10–20 min, depending on the level of detail required on supplement use from participants. The survey was designed using Qualtrics XM survey software. Survey dissemination was primarily online through social media as well as some in-person recruitment at the Sport Ireland Institute with elite athletes. Additional athletes were recruited through personal and professional contacts within private sports clubs and Queen’s University Sport department. Athletes self-identified as either “elite” or “amateur” at the outset of the survey. Inclusion criteria for survey participation were adults aged ≥18 years who compete in any sporting discipline. All complete survey responses were included in the data analysis.

### 2.2. Survey Questions

The survey was designed to be an anonymous, online, self-completion questionnaire. The survey involved 31 questions which were tailored to athletes who self-identified as either elite or amateur and based on answers to previous questions. Questions were partly based on earlier published questionnaires referencing Wardenaar et al. 2017 [16] and Vento and Wardenaar 2020 [17]. A number of questions relating to athlete characteristics were partly based on those asked in a currently unpublished study as part of a PhD thesis at the University of Limerick. Other questions were developed originally to meet the aims of the study. The scope of the survey questions asked included demographics, athletes’ knowledge of BFSs, current BFS intake (including brand name, supplement name, frequency of use, and dosage), reasons for using BFSs, as well as other attitudes and perception questions about BFS use as athletes, including perception of risks associated with use. The full questionnaire and battery of questions included can be found in Supplementary Materials section of this paper.

### 2.3. Data Analysis

Results pertinent to FS and BFS intake, sources of information about BFS, and the types of risks believed to be associated with BFS use are presented descriptively as frequencies and percentages of the survey cohort. Chi-square tests were performed to determine differences in athlete characteristics between supplement users and non-users, where significant results were considered = <0.05. Perceived level of risk associated with BFS use was determined using a sliding Likert scale (1 = not risky at all, 10 = very risky), and results from elite and amateur athletes were reported as means and standard deviation. Differences in perceived level of risk associated with BFS depending on the source of information used were determined by an independent t-test, where significant results were considered *p* = ≤0.05. 

## 3. Results

### 3.1. Characteristics

A total of *n* = 217 complete survey responses were recorded, with *n* = 55 respondents identifying as elite athletes and *n* = 162 as amateur athletes. Athlete characteristics are presented in Table 1 and indicate a relatively even split of males and females for both elite and amateur athletes. Most elite athletes were aged 18–24 years, whereas a more even split between age brackets was noted among amateur athletes; however, this was not statistically significant. Elite athletes mainly competed at international championship level, while most amateur athletes competed at club level either regionally, nationally, or at community games. A variety of primary competitive sports were captured, with a large proportion of amateur athletes competing in a form of Gaelic games (Gaelic football, hurling, camogie). Amateur athletes made up around three quarters (74%) of FS users in this cohort, with elite athletes making up around one quarter (26%). Differences in athlete characteristics between supplement users and non-users are presented in Table 1 and indicate that type of athlete, i.e., elite or amateur, was the only statistically significant differentiating factor between FS users and non-supplement users (*p* < 0.01). Overall, more amateur athletes took part in the study, which may suggest the significant result pertaining to “athlete type”. There were no other significant characteristic differences between supplement users and non-supplement users within the study cohort. 

### 3.2. Types of FS and BFS Used by Athletes

**Figure 1:**
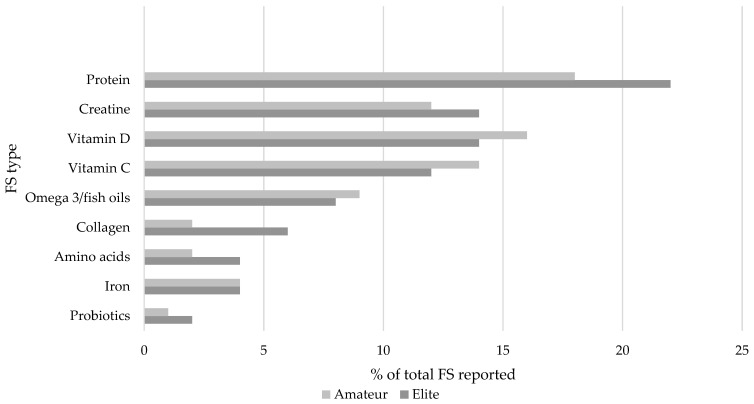
Most reported FSs used by elite and amateur athletes in the survey cohort.

**Figure 2:**
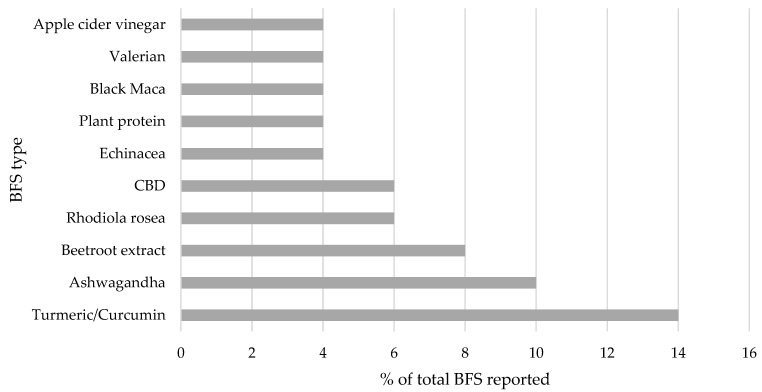
The most reported BFSs by amateur athletes only.

The most frequently reported types of FS are presented in Figure 1, suggesting that “Protein”, “Vitamin D”, and “Vitamin C” were most popular among both elite and amateur athletes. Overall, current BFS use was reported by 16% of the cohort (*n* = 32 amateur, *n* = 2 elite), with elite athletes not disclosing any further detail on BFS names, frequency of use, dosage, or reason for use. Figure 2 presents the most popular types of BFS reported by amateur athletes among a wide variety of products reported, and suggests “Turmeric/Curcumin”, “Ashwagandha”, and “Beetroot extract” were the most frequently reported. Other BFSs which were highly ranked by respondents included, “Chamomile”, “Beetroot extract”, and “Cannabidiol/CBD”. 

### 3.3. Sources of Information about Botanical Food Supplements

When asked about who/where athletes would source information about BFSs from, high proportions of both elite and amateur athletes reported “dietitian/nutritionist” as a top source (75% elite, 69% amateur), followed by “fellow athletes” (44% elite, 51% amateur), and “internet sources” (38% elite, 43% amateur). Findings suggest that amateur athletes are more likely to source information from their “coach” (40%), a “friend” (14%), or “family member” (12%) in contrast to their elite counterparts, at 26%, 7%, and 7%, respectively.

Athletes were also asked who they would consult with before deciding to take a BFS, with similar findings between elite and amateur athletes, as the most popular response for both was a “registered dietitian/nutritionist” (60% elite, 51% amateur). Almost one-third of both elite and amateur athletes would consult with their “coach” or “doctor” and between 22% (elite) and 29% (amateur) would consult a “pharmacist/chemist” before using a BFS. Between 20% (amateur) and 29% (elite) would refer to a “batch testing/third-party testing organisation” such as Informed Sport (https://sport.wetestyoutrust.com/ Accessed 8 August 2022) before taking a BFS product. While all elite athletes reported different individuals or organizations they would consult with, 11% of amateur athletes said they would not consult with anyone before taking a BFS product.

### 3.4. Perceived Level of Risk Associated with Taking BFS Products

Elite and amateur athletes were asked to report on a scale of 1–10 how risky they felt taking a BFS was as an elite or amateur athlete, with 1 indicating ‘no risk at all’ and 10 indicating ‘very risky’. The overall perceived level of risk among all survey respondents was 3.97 (SD 2.5). While elite athletes reported a higher mean level of risk, at 4.8 (SD 2.5), than amateur athletes (mean 3.68 (SD 2.4)), this was not statistically significant (*p* = >0.05). When separated by the sources of information used by all athletes, significant differences in the perceived risk level were observed (Table 2). Specifically, both elite and amateur athletes who reported using internet sources had a significantly lower level of perceived risk than those who did not seek information from this source (*p* = 0.03). Similarly, athletes who sourced information about BFSs from fellow athletes reported a lower level of perceived risk than those who did not seek information from this source (*p* = 0.02). Responses about the types of risks believed to be associated with BFS use differed between elite and amateur athletes. 

The most frequently reported risk among elite athletes was “may cause a positive doping test” (69%), whereas this was reported by 41% of amateur athletes. The top reported risk among amateur athletes was “adverse reactions due to interaction with other medication/supplements” (47%). “Adverse side effects” was reported by 46% of amateur athletes but was less of a concern among elite athletes, with only 27% reporting this. A similar proportion of elite (18%) and amateur (17%) athletes reported that they “did not believe there are any risks associated with the use of BFS”. Additionally, all athletes could report “other” risks they believed to be associated with BFS that were not provided in the list of options. Some examples of “other risks” reported by elite and amateur athletes included “[BFS] are not regulated–no idea how much of the active ingredients [there are] or if there are contaminants [present]” (elite athlete); “[BFS use] can create bad habits regarding skipping meals” (amateur athlete); “Some may cause problems, but not what I take” (amateur athlete).

## 4. Discussion

The results of this study suggest that intake of FSs among elite and amateur athletes living on the island of Ireland is common, with almost three-quarters (74%) of amateur athletes and one-quarter (26%) of elite athletes reporting use. However, BFS intake was reported in detail by amateur athletes only with overall intake being relatively low (16% of cohort currently using BFSs). There were *n* = 2 elite athletes who were currently using BFSs but did not disclose any further information on their intake. The most common type of BFS reported by athletes was turmeric/curcumin products. Existing evidence suggests the potential benefits of curcumin use in the reduction in muscle damage, for increased muscle performance, and anti-inflammatory effects [18] among the athletic population, and this result is therefore unsurprising. Ashwagandha use was reported among some amateur athletes in this cohort, a supplement which has been highlighted as a potential cause for concern among athletes within the testing pool as it can have testosterone boosting effects [19]. Additionally, products containing beetroot extract were also reported by amateur athletes. The products reported are mainly marketed to provide adequate dietary nitrate requirements as well as exercise performance benefits but are also approved for use by Informed Sport. 

Protein and Vitamin D supplements were among the most reported supplements in this study, which is comparable with studies investigating FS use in endurance cyclists, runners, triathletes, wheelchair rugby players, and elite footballers [8,20,21]. Vitamin C products were less prevalent among existing athlete studies [3], but findings from the present analysis in relation to the frequency of Vitamin C intake could be related to the time of data collection occurring during the COVID-19 pandemic and the role of Vitamin C in supporting the immune system (Article 14 of Regulation EC 1924/2006). Intriguingly, Omega 3 supplements were only used by 10% of elite athletes and 8% of amateur athletes, despite there being compelling evidence for the benefits of Omega 3 consumption and/or supplementation on athlete recovery [22,23]. 

When examining the sources of information used by athletes, differences between elite and amateurs were observed. Although dietitians/nutritionists were reported as the most common source of information about BFSs among athletes, results suggest that amateur athletes more frequently reported that they would source information from their coach, a fellow athlete, or a friend or family member. This may be explained by the types of sports played by the cohort of amateur athletes, as a high proportion played Gaelic games (football, camogie, hurling), where access to professional nutrition advice at the amateur level is uncommon. Renard et al. (2022) recently reported that elite and sub-elite Gaelic football players have poor sports nutrition knowledge, which may negatively affect their dietary practices, and that these athletes may benefit from nutrition education interventions [24]. Therefore, coaches and fellow athletes may be the only comparable source of information for amateur athletes to the counterparts such as registered dietitians and nutritionists available to elite athletes on the island of Ireland. Other comparable studies have found that a relatively large range of 19–92% of athletes, including amateur and professional rugby players and National Collegiate Athletics Association (NCAA) Division I athletes, reported sourcing FS information from nutritionists/dietitians [25,26], which may be indicative of the level of professional nutrition advice available to athletes at differing levels of success and competition.

While all elite athletes reported that they would consult with at least one of the listed sources of information, a small proportion of amateur athletes reported they would not consult with anybody, which may reflect a reduced level of concern in relation to BFS use among this group with regards to positive doping testing as well as other risks such as adverse side effects. Indeed, this was reflected in the amateur athletes’ perceived level of risk reported via the sliding Likert scale, which was slightly lower than that reported by elite athletes. This may be because amateur athletes on the island of Ireland, except for those playing in the senior Gaelic football championship, are not subject to doping tests, or it may point to an overall lack of knowledge regarding the potential risks associated with their use. Given that BFS intake is not well-quantified in research in general and specifically among the athletic population, there is a potential lack of knowledge across the board from nutrition practitioners through to coaches and athletes regarding risks associated with use and their lack of substantiated nutrition and health claims. Indeed, a study of sports professionals indicated that coaches, although they are often a main source of information for amateur athletes, are not viewed by other sports professionals as having the ultimate expertise in the area of sports FSs, and only 42% of coaches were confident in their knowledge of sports FSs (self-reported) [27]. Indeed, across the professionals assessed, confidence in being knowledgeable about FSs was lower than their self-reported knowledge on general sport nutrition and therefore emphasizes that FS and BFS education among athletes and coaches is warranted, as they are regarded as a specific group of food products which carry a certain degree of risk for athletes consuming them. Therefore, educational nutrition interventions could have benefits for coaches and athletes alike and may result in safer and more effective use of FSs and BFSs in sport as well as strong advice for athletes, where possible, to seek information about FSs and BFSs from a sports nutrition expert.

Those who sourced information from their coach, from internet sources, and/or fellow athletes reported a lower perceived risk level associated with BFS use. Internet sources such as social media platforms have been highlighted in other studies as a common source of information among female amateur athletes, who also reported the unreliability of information from this source as a concern [27]. Given that a substantial proportion of elite and amateur athletes were sourcing information from the internet about BFSs, and indeed an even higher number of amateur athletes were sourcing information from fellow athletes, friends, or family, an education intervention may be a beneficial way of improving BFS knowledge among amateur athletes in particular and preventing BFS misuse, adverse side effects, and potential doping violations [6,25].

This study had several strengths, including that it is, to our knowledge, the first time BFS use specifically has been investigated within a cohort of both elite and amateur athletes living on the island of Ireland. The overall survey collected data on a wide variety of areas relating to BFS use including consumer knowledge, attitudes, sources of information, and perceptions of risks associated with BFS use. Inherently due to the recruitment and sampling strategy of targeting elite and amateur athletes living on the island of Ireland aged ≥18 years, there is an introduction of bias in this study, specifically through the use of social media as a means of recruitment. Investigating the mean perceived level of risk associated with BFS use in this cohort using a ten-point Likert scale may not be the most appropriate predictor of risk, as only *n* = 2 elite athletes reported that they currently use BFS, and therefore, other predictors should be considered in future studies. Additionally, information on athletes’ perception of the credibility of the sources of information they use for BFSs was not investigated. 

## 5. Conclusions

Intake of any FS by elite and amateur athletes on the island of Ireland is common; however, BFS use is relatively low, though this may be likely to grow with an increase in desire among consumers to seek “natural” health products to take control of their own health. The most common FSs reported by athletes on the island of Ireland were protein, Vitamin D, and Vitamin C, whereas the most reported BFSs were Turmeric, Curcumin, Ashwagandha, and Beetroot extract. This study showed that amateur athletes are more likely to source information from less credible sources in comparison to their elite counterparts. This may have potential adverse implications for such amateur athletes, e.g., Gaelic games players, who are included within the doping testing pool in Ireland. These amateur athletes also believed there was a lower risk associated with BFS use overall. Nutrition-based education interventions would be beneficial for athletes as well as practitioners to ensure that competitive athletes are getting the maximum benefits from their nutritional program, with minimized risk of doping violations and other adverse effects.

## Figures and Tables

**Table 1:** A descriptive comparison of athlete characteristics between any supplement users and non-supplement users in the survey cohort.

	Any Supplement User * *(n* = 129)		Non-Supplement User *(n* = 88)		Chi-Square Test
Characteristics	*N*	%	*N*	%	*p*-Value
Athlete type					
Elite	33	27	22	25	<0.01
Amateur	96	74	66	75	
***Sex***					
Male	68	53	43	49	0.43
Female	61	47	44	50	
Other	-	-	1	1	
Age (years)					
18–24	43	33	41	47	0.22
25–30	36	28	21	24	
31–39	32	25	17	19	
≥40	18	14	8	9	
Highest achievement at competition level					
Club level (regional, national, community games)	65	50	50	57	0.46
National championships	28	22	13	15	
International championships	35	27	23	26	
World Medallist	1	0.8	2	2	
Type of sport					
Athletics and triathlon	34	26	16	18	0.29
Gaelic games	36	28	27	31	
Outdoor field sport	18	14	23	26	
Indoor court sport	3	2	3	3	
Cycling	9	7	6	7	
Swimming/Para swimming	2	2	1	1	
Rowing	7	5	5	6	
Other	20	16	7	8	

* Any supplement user refers to anyone who reported intake of FSs or BFSs, including those who use them both concurrently *(n* = 19 participants). * *p*-value derived from a Pearson Chi-Square test where significant results were considered to be *p* < 0.05. Rounding may have resulted in values slightly below or above 100.

**Table 2:** Differences in mean perceived level of risk associated with BFS use between users and non-users of specific sources of information on BFSs.

Source of Information	Would Use This Source? Yes/No	Total Athletes Reported (%)	Mean Level of Perceived Risk from 1–10 (SD)	*p*-Value
Coach	Yes	38	3.5 (2.0)	0.05
	No	62	4.2 (2.7)	
Doctor	Yes	26	4.5 (2.3)	0.07
	No	74	3.8 (2.3)	
Registered Dietitian /Nutritionist	Yes	71	4.1 (2.4)	0.18
	No	29	4.1 (2.4)	
Internet sources	Yes	42	3.5 (1.9)	0.03
	No	58	4.3 (2.7)	
Fellow athlete	Yes	50	3.6 (2.2)	0.02
	No	50	4.4 (2.6)	
Family member	Yes	10	3.5 (2.0)	0.32
	No	90	4.0 (2.5)	
Friend	Yes	11	3.7 (2.4)	0.56
	No	89	4.0 (2.5)	
Influencer	Yes	2	3.5 (2.1)	0.78
	No	98	4.0 (2.5)	

Self-reported perceived level of risk was reported on a scale of 1–10 (1 being no risk at all and 10 being very risky).

## Data Availability

The data presented in this study are available on request from the corresponding author. The data are not publicly available due to privacy and ethical reasons. Data on questions asked in the study survey are available as Supplementary Materials.

## Supplementary Materials

The following supporting information can be downloaded at: https://www.mdpi.com/article/10.3390/ijerph20136244/s1, Full athlete survey Athlete survey.
