# Supplementary material for: Athletes Perceived Level of Risk Associated with Botanical Food Supplement Use and Their Sources of Information
Source: Int J Environ Res Public Health. 2023 Jun 28;20(13):6244. doi: 10.3390/ijerph20136244 (PMC10341873; doi:10.3390/ijerph20136244)
Supplement: Supplementary file 1 [file ijerph-20-06244-s001.zip › ijerph-2390402-supplementary.pdf]

Q1. Where do you currently live? (Multiple choice single answer)
Northern Ireland

Republic of Ireland

Other

(If ‘Other’ is selected, the survey will skip forward to the end)

Q2. Are you..? (Multiple choice single answer)
Male

Female

Other

Prefer not to say

Q3. What age are you? (Multiple choice single answer)
18-24 years old

25-30 years old

31-39 years old

40+ years old

Q4. Are you an elite athlete or amateur athlete? (Multiple choice single answer)
Elite athlete

Amateur athlete

Q5. What is the highest level of elite sport you have competed at? (multiple choice single answer)
(Display this question if ‘elite athlete’ is selected)

Club level – regional

Club level – national

Community games

National championships medalist

International athlete – European

International athlete – World

Medalist at World Championships/Olympic Games

Other (please specify)

____

Q6. What is the highest level of amateur sport you have competed at? (multiple choice single answer)
(Display this question is 'amateur athlete' is selected)

Club level – Regional

Club level – National

Community games

National championships

International championships

Other (please specify)

____

Q7. What is your primary sporting event? (Drop down list single answer)
(Display this question if 'elite athlete' is selected)

Athletics

Badminton

Basketball

Bob Skeleton

Boxing

Canoeing

Cricket

Cycling

Diving

Golf

Gymnastics

Hockey

Horse Sport

Judo

Mixed Martial Arts

Modern Pentathlon

Netball

Para Athletics

Para Badminton

Para Cycling

Para Swimming

Rowing

Rugby

Sailing

Soccer

Surfing

Swimming

Taekwondo

Tag rugby

Tennis

Triathlon

Prefer not to say

Other

Q8. What is your primary sporting event? (Free text entry)
(Display this question if ‘other’ is selected in Q6)

____

Q9. What is your primary sporting event? (Drop down list single answer)
(Display this question if ‘amateur athlete’ is selected)

Athletics

Badminton

Basketball

Bob Skeleton

Boxing

Camogie

Canoeing

Cricket

Cycling

Diving

Gaelic football

Golf

Gymnastics

Hockey

Horse Sport

Hurling

Judo

Mixed Martial Arts

Modern Pentathlon

Netball

Para Athletics

Para Badminton

Para Cycling

Para Swimming

Rowing

Rugby

Sailing

Soccer

Surfing

Swimming

Taekwondo

Tag rugby

Tennis

Triathlon

Prefer not to say

Other

Q10. What is your primary sporting event? (Free text entry)
(Display question if 'other' is selected in Q8)

____

Q11. Botanical food supplements are food supplements that contain botanical preparations or ingredients.

If you are not sure what botanical food supplements are, we have provided some information below:

Botanicals are either the whole, part, fragmented or cut parts of plants, algae, lichen or fungi. Botanical preparations are obtained by processing the botanicals in some way e.g. pressing, squeezing, extraction, fractionation, distillation, concentration, drying up, and fermentation. The final product can be in powdered (comminuted) form, tinctures, extracts, essential oils, expressed juices, and processed exudates.

More information is available here: https://www.efsa.europa.eu/en/topics/topic/botanicals

Here are some examples of botanical food supplements you may have seen advertised or on sale:

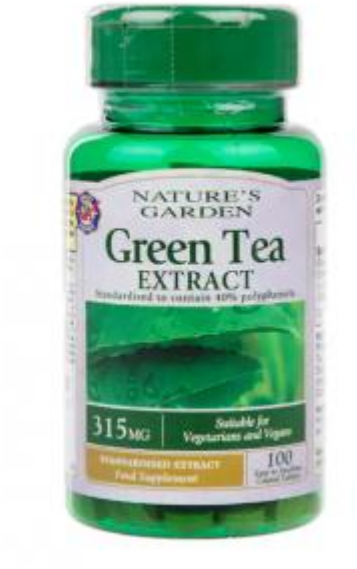

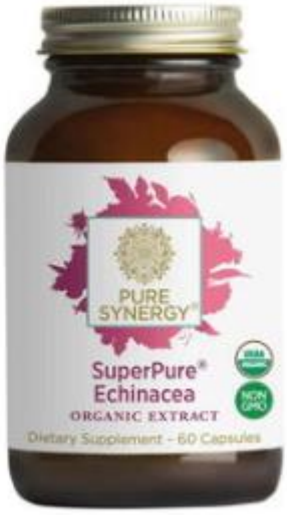

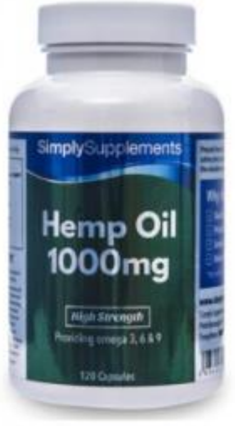

Here are some more examples of botanical food supplements. Have you heard of or seen any of these before? Tick all that apply (Multiple choice multiple answer)

Rhodiola Rosea

Ginseng

Cannabidiol (CBD)

Green Tea Extract

Ginkgo Biloba

Cordyceps Sinensis

Ginger

Uncaria Tomentosa

Alpha GPC

Guarana (Paulinia cupana)

Fenugreek

Beetroot extract

Bacopa

Palmithoylethanolamide (PEA) e.g. Levagen

Ashwagandha

Cranberry

Acai berry

Saw palmetto

Echinacea

Milk thistle

Black cohosh

St. John’s Wort

Chamomile

Peppermint

Valerian

Yohimbe

Cocoa beans

Kola nuts

Yerba mate

Guayusa

Yaupon holly

Sea grape

Yellow horse

Joint fir

Popotillo

Country mallow

I have also heard of others (please specify) (free text)

____

Not sure

I have not heard of any of these botanical supplements

Prefer not to say

Q12. Do you currently take any botanical supplements or would you consider taking them in the future? (Multiple choice single answer)

Yes I currently take botanical supplement(s)

No, but I would consider using them in the future

No and I would not consider using them in the future

Prefer not to say

Don’t know

Q13. If you currently take any botanical supplements, please provide some details in the table below - if you do not know some of the information, please fill out what you can.

Note: If you are unsure of whether you take botanicals and do not want to fill out this table, you can go to the next question and upload photos of the supplements you take and give some detail on how often you use them.

Supplement	Supplement Name	Brand name	Frequency of use	Main reason for use	Dose	Dose unit	Is this supplement third party/batch tested?
	e.g. Korean Ginseng	E.g. Holland & Barrett			e.g. 500 or N/A if dose unknown		
Botanical Supplement 1	Free text	Free text	Drop down list single answer: options<br><br>Daily<br>Less than once a week<br>3-5x a week<br>Weekly<br>More than twice daily<br>Twice daily	Drop down list single answer:<br>Options<br><br>Recovery<br>Immunity<br>Brain function/alertness<br>Strength<br>Endurance<br>Power<br>Sleep improvement<br>Performance anxiety<br>Preventing injury<br>Increase energy<br>Support health<br>Fat burner<br>Other<br>Prefer not to say	Free text	Drop down list single answer: Options<br><br>Mg<br>G<br>µg<br>IU<br>Scoop(s)<br>N/A	Drop down list single answer:<br><br>Options:<br><br>Yes<br>No<br>Not sure<br>Prefer not to say

Botanical Supplement 2							
Botanical Supplement 3							
Botanical Supplement 4							
Botanical Supplement 5							

Q14. If you are not sure if there are botanical ingredients within the supplements you take, you can upload images of the supplement product here.

Please note: Please include the ingredients list in the photo you upload.

Please skip this section if you have already completed the previous table on botanical supplement use.
(Display this question is 'Yes I currently take botanical supplement(s) OR 'Don't know' is selected in Q12)

Choose file to upload

Q15. Please give more detail about the supplement in the photo you have uploaded in the table below

Supplement	Dose	Dose Unit	Frequency of use	Main reason for use	Is this supplement third party/batch tested?
	e.g. 500 or N/A is dose unknown				
Supplement	Free text	Drop down list single answer: Options<br><br>Mg<br>G<br>µg<br>IU<br>Scoop(s)<br>N/A	Drop down list single answer: Options<br><br>Daily<br>Less than once a week<br>3-5x a week<br>Weekly<br>More than twice daily<br>Twice daily	Drop down list single answer: Options<br><br>Recovery<br>Immunity<br>Brain function/alertness<br>Strength<br>Endurance<br>Power<br>Sleep improvement<br>Performance anxiety<br>Preventing injury<br>Increase energy<br>Support health<br>Fat burner	Drop down list single answer Options<br><br>Yes<br>No<br>Not sure<br>Prefer not to say

				Other<br>Prefer not to say	

(Display this question is ‘Yes I currently take botanical supplement(s) OR ‘Don’t know’ is selected in Q12)

Q16. If you are not sure if there are botanical ingredients within the supplements you take, you can upload images of the supplement product here.

Please note: Please include the ingredients list in the photo you upload.

Please skip this section if you have already completed the previous table on botanical supplement use.

(Display this question is ‘Yes I currently take botanical supplement(s) OR ‘Don’t know’ is selected in Q12)

Choose file to upload

Q17. Please give more detail about the supplement in the photo you have uploaded in the table below

(Display this question is ‘Yes I currently take botanical supplement(s) OR ‘Don’t know’ is selected in Q1)

Supplement	Dose	Dose Unit	Frequency of use	Main reason for use	Is this supplement third party/batch tested?
	e.g. 500 or N/A is dose unknown				
Supplement	Free text	Drop down list single answer: Options<br><br>Mg<br>G<br>µg	Drop down list single answer: Options<br><br>Daily<br>Less than once a week<br>3-5x a week<br>Weekly	Drop down list single answer: Options<br><br>Recovery<br>Immunity<br>Brain function/alertness<br>Strength	Drop down list single answer Options<br><br>Yes<br>No<br>Not sure<br>Prefer not to

		IU<br>Scoop(s)<br>N/A	More than twice daily<br>Twice daily	Endurance<br>Power<br>Sleep improvement<br>Performance anxiety<br>Preventing injury<br>Increase energy<br>Support health<br>Fat burner<br>Other<br>Prefer not to say	say

Q18. If you are not sure if there are botanical ingredients within the supplements you take, you can upload images of the supplement product here.

Please note: Please include the ingredients list in the photo you upload.

Please skip this section if you have already completed the previous table on botanical supplement use.

(Display this question is 'Yes I currently take botanical supplement(s) OR 'Don't know' is selected in Q11)

Choose file to upload

Q19. Please give more detail about the supplement in the photo you have uploaded in the table below

(Display this question is 'Yes I currently take botanical supplement(s) OR 'Don't know' is selected in Q11)

Supplement	Dose	Dose Unit	Frequency of use	Main reason for use	Is this supplement third party/batch tested?

	e.g. 500 or N/A is dose unknown				
Supplement	Free text	Drop down list single answer: Options<br><br>Mg<br>G<br>µg<br>IU<br>Scoop(s)<br>N/A	Drop down list single answer: Options<br><br>Daily<br>Less than once a week<br>3-5x a week<br>Weekly<br>More than twice daily<br>Twice daily	Drop down list single answer: Options<br><br>Recovery<br>Immunity<br>Brain function/alertness<br>Strength<br>Endurance<br>Power<br>Sleep improvement<br>Performance anxiety<br>Preventing injury<br>Increase energy<br>Support health<br>Fat burner<br>Other<br>Prefer not to say	Drop down list single answer Options<br><br>Yes<br>No<br>Not sure<br>Prefer not to say

Q20. Does your botanical supplement use change prior to an important competition? (Multiple choice single answer)
(Display this question is 'Yes I currently take botanical supplement(s) OR 'Don't know' is selected in Q11)

No it stays the same

Yes I take them more often

Yes I stop taking them

Yes I increase the dose

Yes I decrease the dose

Other (please specify)

____

Q21. Why would you choose a botanical supplement over food sources? Tick all that apply (Multiple choice multiple answer)
(Display this question is ‘Yes I currently take botanical supplement(s) OR ‘Don’t know’ OR ‘No, but I would consider using them in the future’ is selected in Q11)

Convenience of taking a supplement

I don’t think food would have the same effect

I am not sure of what food to eat to get the same nutrients/benefits

I was recommended the supplement by a professional i.e. dietitian, nutritionist or coach

I would not consider using botanical supplements instead of food sources

Other (please specify)

____

Prefer not to say

Q22. Have you heard/seen any of the following information sources promoting botanical food supplements? Tick all that apply (Multiple choice multiple answer)
Tiktok

YouTube

Podcast

Twitter

Instagram

Other social media platform (please specify)

____

Magazine/internet article

Supplement brand website

Other (please specify)

____

Prefer not to say

Don’t know

Q23. Who/where would you go to source information about botanical food supplements/any supplements before you start to use them? Tick all that apply (Multiple choice multiple answer)

Coach

Doctor

Dietitian or nutritionist

Internet sources

Fellow athlete

Family member

Friend

Influencer/celebrity endorser

Don't know

Prefer not to say

Other (please specify)

____

Q24. Where do you purchase, or where would you consider purchasing your botanical supplements from? Tick all that apply (multiple choice multiple answer)
(Display this question is 'Yes I currently take botanical supplement(s) OR 'Don't know' OR 'No, but I would consider using them in the future' is selected in Q11)

Online/Internet

In a store

From a coach

Herbal/Botanical Remedy practitioner

All of the above

Other (please specify)

____

Prefer not to say

Q25. On a scale of 1 to 10, 1 being no risk at all and 10 being very high risk – how risky do you think taking botanical supplement is as an elite athlete? (Likert scale)
(Display this question if 'elite athlete' is selected)

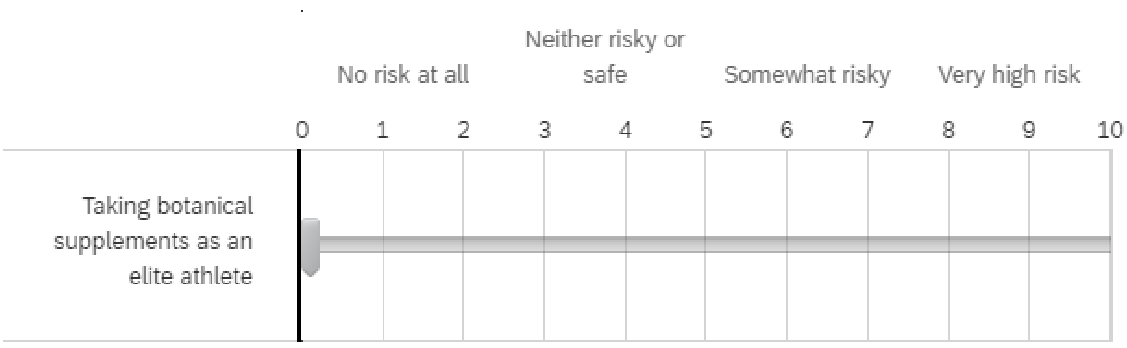

Q26. On a scale of 1 to 10, 1 being no risk at all and 10 being very high risk – how risky do you think taking botanical supplement is as an amateur athlete? (Likert scale)
(Display this question if 'amateur athlete' is selected)

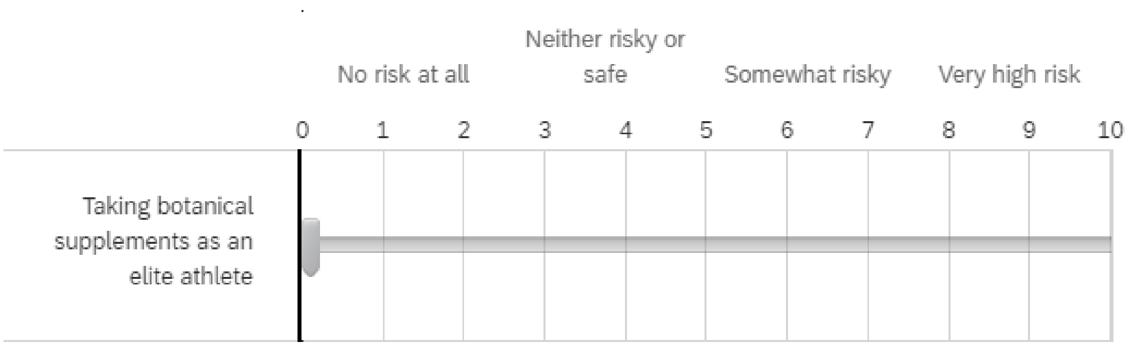

Q27. What risks do you think may be associated with botanical supplement use? Tick all that apply (Multiple choice multiple answer)
Adverse side effects

Adverse reaction due to interaction with other medication/supplements

It may cause a positive doping test for elite athletes

Other (please specify)

____

I don't believe there any risks associated with the use of botanical supplements

Q28. Do you agree with the following statements

a) The purity and safety of all supplements is tested before sale. (Multiple choice single answer)
Agree

Disagree

Not sure

b) Supplement labels may contain false or misleading information.
Agree

Disagree

Not sure

Q29. Who would you consult with before deciding to take a botanical supplement(s)? Tick all that apply (Multiple choice multiple answer)
Coach

Doctor

Registered Dietitian or Nutritionist

Pharmacist/Chemist

Fellow team mate or athlete

Third Party Testing e.g. Informed Sport

World Anti-Doping Agency (WADA) website

Other (please specify)

____

I do not feel that I would need to consult with anyone before taking a botanical supplement

Prefer not to say

Q30. Do you take any other food supplements are part of your regimen as an athlete? (Multiple choice single answer)
Yes

No

Don’t know

Prefer not to say

Q31. If you take any other food supplements are part of your regimen as an athlete, please provide more detail of the products you use in the table below
Please turn your phone horizontal to view the full table.
(Display this question if ‘Yes’ or ‘Don’t know’ is selected in Q28.

Supplement	Supplement name<br>e.g. Vitamin C	Brand name<br>e.g. Tesco	Frequency of use	Main reason for use	Dose<br>e.g. 200 or N/A if dose unknown	Dose unit
Supplement 1	Free text	Free text	Drop down list single answer: Options<br><br>Daily<br>Twice daily<br>More than twice daily<br>Less than once a week<br>3-5x a week<br>Weekly	Drop down list single answer: Options<br><br>Recovery<br>Prevent illness/immunity purposes<br>Support health<br>Brain function/alertness<br>To fulfil part of my dietary requirements<br>Endurance<br>Power<br>Sleep improvement<br>Performance anxiety<br>To prevent injury<br>Increase energy<br>Increase muscle mass<br>Strength<br>Fat burner<br>Other<br>Prefer not to say	Free text	Drop down list single answer: Options<br><br>Mg<br>G<br>Mg<br>IU<br>Scoop(s)<br>Tab/Effervescent<br>Ml<br>N/A

Supplement 2						
Supplement 3						
Supplement 4						
Supplement 5						
